# Annotations

**Published:** 1893-01-21

**Authors:** 


### ANNOTATIONS.

Cherryhinton to the Rescue.

DOES any benighted reader ask where Cherryhinton is? All the roads in Great Britain lead to Cherryhinton if you pursue them in the right direction, and they come upon it in Cambridgeshire, quite close to the University of Cambridge. A short time ago a patient suffering from typhoid fever was removed from Addenbrook's Hospital to a nurse's cottage at Cherryhinton. The probability is that the patient was convalescent, and was taken there for change of air. But Cherryhinton was not to be imposed upon. The inhabitants got wind of the affair, and were speedily up in arms. They met in a body, and passed a resolution, in Cherryhinton grammar, calling upon the public authorities to remove the unfortunate patient on pain of penalties of the most ultimate and tremendous character. The doctor, who was first appealed to, laughed at them; and, joining in the doctor's laugh, we would beg the Cherryhinton folk, and all whom it may concern, to acquire a little more knowledge wherewith to temper their zeal. Typhoid fever, like cholera, is not infectious, except by means of the excreta of the affected patient. In this it differs from small-pox, scarlatina, measles, and the like, and is therefore practically a source of no danger, except to uncleanly or careless persons. The local newspaper took the part of the ungrammatical memorialists. But the editor ought to have known better. Infectious diseases often raise important public questions, and the editors of newspapers ought to recognise it as part of their duty to understand the general nature of such diseases, and to be able to distinguish between those that differ. In cases where they do not know they might, without loss of dignity and with much gain of usefulness, consult the local practitioner. We notice this village scare in order that we may warn other persons who lie a little off the main lines of civilisation against making themselves similarly ridiculous.

Pasteur and Jenner.

MR. WILLIAM TEBB, the leader of the Anti-Vaccinationists, whose letter we print elsewhere, takes occasion, from the celebration of M. Pasteur's seventieth birthday, to make another appearance in his *rôle* of the prophet Jonah against an unbelieving generation. The public has to choose between such men as Jenner, Pasteur, and Lister, on the one hand, and Mr. Tebb, with others much less well-informed and much less capable of judging than even he, on the other. The public, much to Mr. Tebb's disgust, manifestly prefers Pasteur and Lister, and even Jenner: and not the British public only, but the Continental and the American. This is a poor result of a passionate propaganda. Statistics are certainly against Mr. Tebb, and common experience is an overwhelming adversary. Medical statistics may not be more trustworthy than other statistics. We believe, however, that among the various classes of statistics, those of medicine will rank with the highest. It is Mr. Tebb's custom to answer statistics by counter-statistics. This is a questionable sort of game to play at. Mr. Tebb doubts our statistics—we doubt his. It is as well to point out, however, that the witnesses on the side of vaccination are much more numerous and much more competent than those on the other side, and therefore much more credible. But common experience seldom deceives, and common experience tells this convincing tale, that whereas forty years ago one could hardly walk through a single street in town or village without meeting one or more persons repulsively pitted by small-pox, to-day it is almost impossible to find a person anywhere under forty years of age who is pitted with small-pox marks at all. So much for Jenner! As for Pasteur: will anybody deny that in finding out the microbial nature of infectious diseases generally the French scientist has advanced the science of medicine more than it had before been advanced by any single individual in any age? The art of medical treatment he may not have advanced so much, though even that more competent judges than Mr. Tebb think he has advanced amazingly. Time, at any rate, will show. But that the knowledge of the causes of disease, the science which is the foundation of the art, has been wonderfully advanced, not even Mr. Tebb himself ventures to deny. It is quite proper that Mr. Tebb and his associates should question the utility of vaccination, or of any other method of prophylaxis or cure which it pleases them to question; but it is not proper, it is not even fair, that in carrying out their propaganda they should seek to throw discredit on what they cannot deny to be admirable work in order that they may discredit the character and reputation of the worker. It is hitting below the belt, Mr. Tebb, and it serves no other purpose than that of discrediting yourself and your associates.

Medical Missionaries.

THE Edinburgh Medical Missionary Society has sent to us a copy of the Life of the Rev. John Lowe, F.R.C.S.E. Dr. Lowe was a medical man and a missionary all his adult life. He was a man of science and a man of piety; a surgeon and a saint; a physician and a preacher of religion. Some undoubtedly clever people shrug their shoulders at such a combination. They think, or affect to think, that the saint and the scientist cannot exist in the same person. Granted the saintship, they say, and the science cannot be worth much. Is this a really intelligent attitude of mind? What is saintship? It is in ultimate analysis but a profound view of moral duty, and a highly imaginative, but sober realisation of ideas and ideals of which every thinker must at least admit the possibility. True science has nothing whatever to do with absolute denials and universal impossibles. It seems to us that the Christian religion, as understood and taught by its wisest exponents, and more especially by those of its exponents who have had a thorough training in medicine, is eminently adapted to produce in worn-out civilisations just that revivification, and in untaught and savage races just that intellectual stimulus, enlightenment, and expansion which a universal science looks for as the final result of its work. The races of men must go forward or backward. What is so truly "forward" as mental discipline and moral purification? And what single study is so calculated to produce both these, as a deep and practical training in the Christian faith? It is our conviction that just as Moses was the parent of a new civilisation, as well as of a new religion, so is Christianity, in its essence, the intellectual seed-bed from which not only a better religion, but a higher civilisation and a truer and more beautiful science have sprung. Christianity, at bottom, is scientific, and is not so much the twin as the *bona fide* parent of modern science. The missionary, medical or otherwise, who carries an intelligent Christianity, carries a true science into the remotest corners of the earth.

